# Knowledge, attitudes and practice of consumers towards single-use plastics at Korle-Gonno, Ghana

**DOI:** 10.1371/journal.pone.0327374

**Published:** 2025-07-09

**Authors:** Elijah Kwasi Peprah, Samuel Agyabeng Boapeah, Roberta Amewor, Christine Tettey, Forgive Awo Norvivor, Doreen Danso, Reginald Eshun, Hope Yaw Attah, Michael Affordofe

**Affiliations:** 1 University of Health and Allied Sciences, Ho-Ghana—Department of Epidemiology and Biostatistics,; 2 Accra School of Hygiene, Accra, Ghana—Department of Environmental Health; University of Mpumalanga, SOUTH AFRICA

## Abstract

**Introduction:**

Plastic pollution, particularly from single-use plastics (SUPs), is an increasing environmental problem, especially for coastal communities dependent on marine ecosystems for their livelihood, food, and recreation. The Korle-Gonno community in Ghana epitomizes this: inadequate waste management systems and heavy reliance on SUPs. This study aimed to explore the drivers of consumer attitudes toward SUPs to inform effective interventions.

**Methods:**

A descriptive cross-sectional study was conducted among 198 residents of Korle-Gonno. The study used a multi-stage sampling method to select participants, targeting adults who had lived in the community for at least five years. Data was collected using a structured questionnaire that assessed socio-demographics, knowledge, concerns, attitudes, and practices regarding SUPs. Scores were categorized into low, moderate, and high levels, and data were analyzed using STATA 17.0. Descriptive and inferential statistics were analyzed, including chi-square tests and logistic regression.

**Results:**

The study showed that 51% of the respondents have low knowledge of SUPs and although they considered SUPs as one of the major contributors to pollution, only 10.1% reported recycling plastics, while most relied on improper methods of plastic disposal. Important predictors of positive attitude towards the reduction of SUPs were: high levels of concern (aOR=2.37, 95% CI = 1.09–5.15) and good perception of environmental impact (aOR=4.59, 95% CI = 2.15–10.83). Those who had lived in the community for more than 20 years were likely to have positive attitudes (aOR=2.45, 95% CI = 1.04–5.77).

**Conclusion:**

SUP pollution in Korle-Gonno is fueled by a lack of knowledge, inappropriate practices, and inadequate infrastructure. While these are major challenges, the community strongly supports regulation and corporate responsibility. Public education, improvement of the recycling system, and promotion of biodegradable alternatives are critical intervention strategies to solve this problem sustainably.

## Introduction

Over recent years, plastic pollution has become one of the most daunting environmental problems, especially in areas where human activities merge with marine ecosystems [1]. Community settings in the coastal areas, which often depend on marine resources for livelihood purposes, are particularly more vulnerable to the presence of plastic waste due to inadequate infrastructures for waste management, making it difficult to handle or dispose of waste effectively [2]. Depending on their application, plastics can be further categorized into general plastics and engineering plastics [3]. Polyethylene (PE), polypropylene (PP), polyvinyl chloride (PVC), polystyrene (PS), polyurethane (PU), and phenolic resin are the major general plastics. Among these, PP and PE are the most common polymers used in daily plastic products, especially single-use and non-degradable disposable products such as plastic packaging and disposable water bottles [4,5].

The United Nations Environment Programme (UNEP) defines single-use plastics (SUPs) as “an umbrella term for different types of products that are typically used once before being thrown away or recycled” including food packaging bottles, straws, containers, cups, cutlery, and shopping bags [6]. Single-use plastic waste originates mainly from two major sources: land-based and water-based activities [7]. The land-based activities include industries, tourism, recreational activities, households, poorly managed mulching film wastes, poorly treated sewage, and unprotected landfills. Water-based activities, cover fisheries, commercial and recreational shipping, and runoff from the adjacent areas. The reliance on single-use plastics such as bags, bottles, and food containers has escalated due to urbanization, population growth, and changing consumer behaviors [8].

Nearly 400 million metric tonnes of plastic are produced annually, which eventually contributes to a high level of environmental pollution globally [9]. The production quantities are estimated to be half of the entire plastic quantity produced while SUPs packaging is the largest portion produced.

Over a quarter, 26%, of these plastics are manufactured in Northeast Asia, followed by North America at 21%, the Middle East at 17%, and Europe at 16% [10]In Africa, the largest use of single-use plastics is for packaging. In countries like Ghana, South Africa, and Nigeria, single-use plastics could account for 60–70% of visible litter on streets reflecting their widespread use, attitude of the consumers, and poor waste management systems [11]. It is estimated that only 14% of packaging waste is currently recycled, while over 80% is disposed of or dumped as litter, of which 40% goes to landfills, 14% is incinerated, and 32% is released into the natural environment in Africa [12].

Ghana a low-middle-income country in West Africa exemplifies this predicament, facing enormous challenges in managing single-use plastics [13]. More than 3,000 metric tons of plastic waste are produced every day and one million tons annually, despite the absence of precise data on total plastic manufacturing and imports [14]. Due to the poor practices and attitudes of the consumer, most single-use plastic products and microplastics find their way into landfills, river bodies, marine environments, and lagoonal environments, where they are poised to impact the health of wildlife, humans, and aquatic life [15,16]. It is therefore not surprising that about 500 kilometers of Ghana’s coastline are littered with trash comprising single-use plastics [17].

The Greater Accra of Ghana is facing a growing challenge as single-use plastics are taking over the city [18]Accra generates over 3,000 metric tonnes of waste every day, and a significant chunk of that is plastic, these are mostly items that are used once and thrown away (Okai, 2020). The impact reaches the coastline too, where plastic waste drifts into the ocean, polluting the water and threatening marine life [19]. While there have been several studies on systems of plastic waste management in Ghana, the drivers of attitudes towards single-use plastics are inadequately understood in coastal communities. This knowledge gap is acutely felt in the coastal regions of Accra, home to residents who directly depend on activities that are affected by marine plastic pollution like Korle-Gonno, however their views and attitudes towards single-use plastics are under-researched. These attitudes, along with their drivers, need to be understood to formulate effective community-based interventions and policies that contribute toward reducing plastic pollution on these susceptible coasts. This study, therefore, seeks to assess the drivers of attitudes towards single-use plastics among consumers in Korle-Gonno in Greater Accra, Ghana.

## Methods

### Study area

Korle-Gonno is a thriving coastal community in Accra, created in the early 1900s around main economic hubs like the Korle Bu Teaching Hospital and the Tuesday Market. However, the excesses of rapid urbanization have resulted in appalling environmental and economic decadence. The housing conditions within the community are characterized by overcrowding and poor environmental quality, including inadequate sanitation facilities. The educational levels are low, with the majority not completing secondary education due to financial constraints. The limited alternative livelihood and vocational training due to the high costs force the youth into informal jobs at meager wages. Korle-Gonno has a population of 27,826 [20].

### Participants and procedure

#### Type of study.

A descriptive cross-sectional study design was conducted to assess drivers of attitudes of consumers towards single-use plastic pollution. This design ensured that the study was conducted simultaneously or over a short period to aid researchers in assessing a snapshot of the population on an issue.

### Sampling

#### Inclusion and exclusion criteria.

The study included only residents who had lived within the community for over five (5) years and were above eighteen (18) years old, and were willing to participate in the study. Residents who have lived in the community for at least five years are likely to have a deep understanding of the community’s dynamics, changes, and long-term trends. This long-term perspective can provide richer and more insightful data on issues surrounding single-use plastics within the coastal communities. Also, by including only participants who are eighteen years or older, the study ensures that the respondents are legal adults and can provide informed consent.

Residents who have lived in the community for less than five (5) years and the population below the age of eighteen (18) years were excluded from the study. This ensures that participants have a stable and informed perspective on community life regarding single-use plastics. Short-term residents may not have sufficient experience to provide meaningful insights into long-term trends and community dynamics. Finally, excluding individuals under eighteen years old ensures that participants are legal adults who can provide informed consent.

#### Sample size determination.

The sample size for the study was calculated using the Cochran formula (1963) to select 198 participants for the study.

#### Sampling method.

This study employed a carefully designed multi-stage sampling approach to investigate single-use plastic pollution at Korle-Gonno. The first stage involved stratifying the Korle-Gonno community into distinct local sections based on geographical boundaries. Within each stratum, a simple random sampling (SRS) technique was employed to select specific households, using household lists obtained from community leaders and local records. This ensured that each household within a stratum had an equal chance of selection.

Thus, while stratified sampling provided structure and representation across different zones, SRS was nested within it to ensure unbiased selection. This layered method enhanced the representativeness and reduced potential selection bias.

The final stage of the sampling involved purposive selection of individuals within the selected households. Specifically, only adults aged 18 years and above who had resided in the community for at least five years were eligible. This criterion was important to ensure that participants had sufficient experience and understanding of local dynamics regarding single-use plastics.

### Data collection tool and procedure

Data collection was conducted by three trained research assistants and the principal investigator using a structured questionnaire to assess knowledge, attitudes and practices of consumers towards single-use plastics at Korle-Gonno. The data collection was done within a month (from 1^st^ July to 31^st^ July, 2024). The question items were pre-tested at the Jamestown coastal community to check for clarity and appropriateness, and to familiarize the data collectors with ethical practices.

The question consisted of six (6) sections;

Socio-demographic characteristics of participants,

Consumers Knowledge on Single-use Plastics,

Consumer Concern about the Harmful Effects of Single-Use Plastics,

Consumer Perception of Single-Use Plastics’ Harmful Effects,

Consumer attitudes towards Single-Use Plastics and

Consumer Disposal Methods of Single-Use Plastics.

Informed consent (verbal consent) was documented through a witness signature confirming that the participants had given consent. Data collectors were trained to maintain confidentiality and respect throughout data collection.

### Measures

In the study, participants’ knowledge levels, concerns, and attitudes were categorized into low, moderate, and high classifications based on their scores derived from a structured questionnaire. Correct or positive responses were allocated a score of one point, while incorrect, neutral, or negative responses were assigned zero points. The total score for each participant within a category was calculated as the sum of individual response scores. Thresholds for classification were determined to group scores into predefined ranges, with low scores representing the lowest third of the range, moderate scores falling within the middle third, and high scores comprising the upper third. Alternatively, a percentage-based criterion was applied, categorizing scores of 40% or lower as low, 41–70% as moderate, and above 70% as high. For Likert-scale items, average response scores were employed, with averages below 2 classified as low, 2–4 as moderate, and above 4 as high. These thresholds were likely established during the study’s design phase and informed by expert judgment, pilot testing, or references to prior research to ensure meaningful and consistent differentiation among participants’ responses. This methodological approach facilitated the classification of data for subsequent statistical analysis and interpretation.

### Data analysis

Data was collected and entered into Google Forms, which allowed for efficient and systematic data entry. After the data was gathered, it was exported to Stata 17.0, a statistical software package used for data management and analysis. Descriptive statistics such as frequencies and proportions were performed for categorical variables, whereas means and standard deviations were computed for continuous variables and are presented in tables and charts. Additionally, percentages were calculated to provide a quantitative summary of the data, making it easier to interpret the findings. Inferential statistics was used for associations and predictors.

### Ethical approval

This study was ethically approved by the University of Health and Allied Sciences Review Committee with the number **(UHAS-REC B.10 [096] 23-24)** and the Unity Committee of Korle-Gonno. Participants were given written informed consent, informed of the study’s purpose, and given code names to conceal their identities. Interview recorders were encrypted, and the data collected was protected. There were no known risks or individual benefits associated with participating in the study.

## Results and findings

### Socio-demographic characteristics of participants

From Table 1, the study involved 198 participants with an average age of 35.89 years, with the majority (61.1%) aged 25–44 years. Females made up 55.6% of participants, and males 44.4%. Regarding education, 43.4% had basic education, 24.2% completed senior high school, and 22.7% had tertiary education. Most participants were single (55.1%), with 37.4% married or cohabiting. Over half (55.1%) lived with family, while 32.8% lived alone. A majority (69.7%) were self-employed. Urban residents comprised 67.7% of participants, while 32.3% lived in rural areas, and 40.9% had lived in their community for over 20 years.

**Table 1:** Socio-demographic characteristics of participants.

Variable	Frequency (n = 198)	Percent (%)
**Age mean (±sd)**	35.89(±14.43)	
<25	52	26.3
25-34	62	31.3
35-44	25	13.0
45+	59	30.0
**Sex**		
Female	110	56.0
Male	88	44.4
**Education**		
None	19	10.0
Basic	86	43.4
SHS	48	24.2
Tertiary	45	23.0
**Marital status**		
Single	109	55.1
Married/cohabitation	74	37.4
Previously married	15	8.0
**State of living**		
Alone	65	33.0
With family	109	55.1
With partner	24	12.1
**Occupation**		
Unemployed	31	16.0
Self-employed	138	70.0
Employed	29	15.0
**Residential area**		
Rural	64	32.3
Urban	134	68.0
**Years in Community**		
5-10	66	33.3
11-15	15	8.0
16-20	36	18.2
Above 20	81	41.0

***Data Source: July 1 to July 31, 2024.***

#### Consumer concern about the harmful effects of single-use plastics.

Regarding concerns about SUP’s role in air pollution from incineration, 31.0% are very aware, and another 13.0% are aware, indicating a strong understanding among some participants. However, 31.3% are either not very aware or completely unaware, showing gaps in knowledge. When it comes to concern about SUP’s impact on air quality and pollution, 54% of respondents express at least some degree of concern, ranging from slightly to extremely concerned. Conversely, 26.0% are not concerned at all. For the impact on waterways, 63.0% show varying degrees of concern, while 22.2% are not concerned. Similarly, concern for marine ecosystems and wildlife is expressed by 72.3% of respondents, though 27.8% remain unconcerned.

#### Consumer perception of single-use plastics’ harmful effects.

Results in (Table 4) revealed that 57.1% of respondents had a good perception of the harmful effects of single-use plastics (SUPs), while 43.0% had a poor perception. Regarding the environmental threat of SUPs, 43.0% viewed them as a significant threat, with 32.3% seeing them as a moderate threat. Specifically, 56.0% of participants considered SUPs a moderate to significant threat to marine life.

**Table 4:** Consumer Perception of Single-Use Plastics’ Harmful Effects.

Variable	Frequency (n = 198)	Percent (%)
**Single-use plastics (SUP) have a significant impact on environmental pollution.**		
No, not at all	44	22.2
Maybe to a small extent	48	24.2
Yes, to a moderate extent	43	22.0
Yes, it’s a significant contributor	59	30.0
I’m unsure	4	2.0
**Believe SUP contributes to blocking drains, and waterways and causing flooding**		
No, not at all	38	19.2
Maybe to a small extent	41	21.0
Yes, to a moderate extent	51	26.0
Yes, it’s a significant contributor	61	31.0
I’m unsure	7	4.0
**SUPs harm marine life and the marine environment**		
No, not at all	47	24.0
Maybe to a small extent	33	17.0
Yes, to a moderate extent	48	24.2
Yes, it’s a significant contributor	62	31.3
I’m unsure	8	4.0
**Litter from SUPs is a major problem on streets and public spaces**		
No, not at all	41	21.0
Maybe to a small extent	39	20.0
Yes, to a moderate extent	50	25.3
Yes, it’s a significant contributor	61	31.0
I’m unsure	7	6.0
**SUPs contribute significantly to air pollution**		
No, not at all	62	31.3
Maybe to a small extent	34	17.2
Yes, to a moderate extent	43	22.0
Yes, it’s a significant contributor	51	26.0
I’m unsure	8	4.0
**Level of threat SUPs pose to the environment**		
No threat at all	11	6.0
A minor threat	29	15.0
A moderate threat	64	32.3
I am unsure	9	5.0
Yes, a significant contributor	85	43.0
**Environmental problem is most worsened by SUPs**		
Air pollution	46	23.2
Clogged drains and flooding	67	34.0
Littering and visual pollution	31	16.0
Damage to marine ecosystems	47	24.0
Unsustainable resource use	7	4.0
**Factors that contribute the most to plastic pollution**		
Irresponsible waste management	47	24.0
Excessive use of single-use plastics	46	23.2
Lack of recycling infrastructure	67	34.0
Limited awareness about the issue	22	11.1
Industrial pollution	12	6.1
Other	4	2.0

***Data Source: July 1 to July 31, 2024.***

#### Consumer perception of single-use plastics’ harmful effects.

#### Consumer practices towards single-use plastics.

The study also examined daily practices regarding plastic use. Most participants (77.0%) bought water and soft drinks in plastic bottles daily, and 70.2% acquired snacks wrapped in plastic films daily. About 59.1% used plastic shopping bags daily, while 63.0% purchased takeaway food in Styrofoam packs daily. Most participants reused plastic items multiple times before disposal, with 49% reusing plastic bottles 2–3 times on average, and 43.0% reusing plastic shopping bags 2–3 times.

#### Consumer attitudes towards single-use plastics.

Attitudes towards SUP pollution revealed that 53.1% of participants recognized SUPs as a significant contributor to plastic pollution in Ghana, and 43.0% viewed SUPs as posing a significant environmental threat. Most participants (81.3%) supported local regulations aimed at reducing SUP consumption, and an overwhelming 90.4% believed the government should implement stricter regulations on SUPs. Additionally, 67.0% strongly agreed that companies should be held responsible for the environmental impact of their SUP products.

#### Consumer Disposal Methods of single-use plastics.

Disposal methods for SUPs varied, with 57.0% of participants discarding plastic bottles in bins, while 26.0% burned them. Only 10.1% reported recycling their plastic bottles. A similar pattern was observed for the disposal of plastic films, bags, and Styrofoam packs. Barriers to reducing SUP consumption were identified, with 35.4% citing the lack of alternatives, and 29.3% noting a lack of awareness about the environmental impact of SUPs. Participants also highlighted challenges such as the high cost of reusable alternatives and the inconvenience of recycling options.

### Bivariate and multivariate analysis of the factors influencing good attitude towards SUPs

Table 8 presents the Bivariate and multivariate analysis of the factors influencing good attitude towards SUPs. The results revealed that marital status of the participants (p = 0.005), living condition (p = 0.014), residential area (p = 0.011), years in community (p = 0.007), perception towards SUPS (p < 0.001) and level of concern for SUPs (p < 0.001). First, marital status emerged as an important factor, with a p-value of 0.005 indicating that whether participants are married or single affects their attitudes toward SUPs. Similarly, living conditions were also found to play a role, as evidenced by a p-value of 0.014, suggesting that the quality of one’s living environment impacts their perspective on sustainability.

**Table 8:** Bivariate and multivariate analysis of the factors influencing good attitudes towards single-use plastic.

Covariates	Attitude towards SUPs		χ^2^(p-value)	cOR (95% CI), p-value	aOR (95% CI), p-value
	Poor 48.9(42.1-55.9)	Good 51.0(44.0–57.9)			
**Age**			1.87(0.600)		
<25	23(44.2)	29(55.8)		Ref.	Ref.
25-34	30(48.4)	32(51.6)		0.85(0.40-1.77),0.658	1.03(0.38-2.81),0.952
35-44	11(44.0)	14(56.0)		1.01(0.39-2.64),0.985	1.74(0.41-7.32),0.452
45+	33(55.9)	26(44.1)		0.62(0.29-1.32),0.220	1.42(0.36-5.61),0.618
**Sex**			0.68(0.409)		
Female	51(46.4)	59(53.6)		Ref.	
Male	46(52.3)	42(47.7)		0.79(0.45-1.38),0.409	
**Education**			2.44(0.486)		
None	11(57.9)	8(42.1)		Ref.	
Basic	39(45.3)	47(54.7)		1.66(0.61-4.53),0.325	
SHS	27(56.3)	21(43.7)		1.07(0.37-3.13),0.903	
Tertiary	20(44.4)	25(55.6)		1.72(0.58-5.08),0.327	
**Marital status**			**10.78(0.005)**		
Single	42(38.5)	67(61.5)		Ref.	Ref.
Married/cohabitation	45(60.8)	29(39.2)		**0.40(0.22-0.74),0.003**	0.38(0.12-1.25),0.111
Previously married	10(66.7)	5(33.3)		**0.31(0.10-0.98),0.046**	0.27(0.06-1.29),0.102
**State of living**			**8.61(0.014)**		
Alone	26(40.0)	39(60.0)		Ref.	Ref.
With family	53(48.6)	56(51.4)		0.70(0.38-1.31),0.270	0.71(0.29-1.72),0.455
With partner	18(75.0)	6(25.0)		**0.22(0.08-0.63),0.005**	0.41(0.09-1.77),0.235
**Occupation**			1.56(0.458)		
Unemployed	12(38.7)	19(61.3)		Ref.	
Self-employed	70(50.7)	68(49.3)		0.61(0.28-1.36),0.229	
Employed	15(51.7)	14(48.3)		0.59(0.21-1.64),0.313	
**Residential area**			**6.45(0.011)**		
Rural	23(35.9)	41(64.1)		Ref.	Ref.
Urban	74(55.2)	60(44.8)		**0.45(0.25-0.84),0.012**	0.63(0.29-1.35),0.235
**Years in community**			0.007*		
5-10	40(60.6)	26(39.4)		Ref.	Ref.
11-15	11(73.3)	4(26.7)		0.56(0.16-1.95),0.361	0.80(0.19-3.43),0.766
16-20	16(44.4)	20(55.6)		1.92(0.85-4.38),0.119	2.52(0.86-7.39),0.093
Above 20	30(37.0)	51(63.0)		**2.62(1.34-5.10),0.005**	**2.45(1.04-5.77),0.041**
**Knowledge regarding the issues of SUPs**			4.86(0.088)		
Low	56(56.0)	44(44.0)		Ref.	Ref.
Moderate	12(35.3)	22(64.7)		**2.33(1.04-5.23),0.040**	1.03(0.38-2.83),0.953
High	29(45.3)	35(54.7)		1.54(0.82-2.89),0.182	0.89(0.39-2.01),0.780
**Perception towards SUPs**			**37.63(<0.001)**		
Poor	63(74.1)	22(25.9)		Ref.	Ref.
Good	34(30.1)	79(69.9)		**6.65(3.54-12.49), < 0.001**	**4.59(2.15-10.83), < 0.001**
**Level of concern for SUPs**			**15.25(<0.001)**		
Low	65(60.2)	43(39.8)		Ref.	Ref.
Moderate	9(56.2)	7(43.8)		1.18(0.41-3.39),0.765	0.62(0.17-2.32),0.482
High	23(31.1)	51(68.9)		**3.35(1.79-6.26), < 0.001**	**2.37(1.09-5.15),0.030**

***Data Source: July 1 to July 31, 202.***

Residential area further highlights this influence, with a p-value of 0.011 indicating that attitudes vary depending on geographic location. Additionally, the length of time participants has spent in their community significantly correlates with their attitudes, as shown by a p-value of 0.007; those who have lived longer in the community tend to have more favorable views on SUPs.

The analysis also underscores the critical importance of perception. Participants’ views about SUPs themselves are profoundly significant, with a p-value of less than 0.001. This suggests that how individuals perceive these practices is crucial in shaping their attitudes. Finally, the level of concern for sustainability also stands out as a key factor, with a similarly low p-value of less than 0.001, indicating that greater concern leads to more positive attitudes.

Adjusting for the confounders, those who have stayed in the community above 20 years are two times more likely to have good attitude towards single use plastics as compared to those who have been there between 5–10 years (aOR=2.45,95% CI = 1.04–5.77). In addition, those who have good perception towards single use plastics are almost five times more likely to have good attitude towards single use plastics as compared to those who have poor perception (aOR=4.59, 95% CI = 2.15–10.83). Finally, those with a high level of concern for SUPs are two times more likely to have good attitude towards single use plastics as compared to those low level of concern (aOR=2.37, 95% CI = 1.09–5.15).

## Discussions

### Knowledge level of consumers on single-use plastics

The results in (Table 2) revealed that over half of the respondents (51.0%) had low knowledge about single-use plastics (SUPs), while 32.3% had high knowledge, and 17.2% had moderate knowledge. Only 36.4% correctly identified SUPs as plastics designed for one-time use, while 65.0% misunderstood this classification. This highlights a significant gap in public awareness of plastic pollution, which aligns with findings from other studies. [21] suggests that increased awareness could drive environmentally friendly behaviors, and [22]link knowledge with pro-environmental attitudes. However, the COVID-19 pandemic has exacerbated reliance on SUPs, as noted by [23], leading to misconceptions about their necessity. [24] also observes that the widespread use of personal protective equipment (PPE) during the pandemic normalized SUPs. This lack of knowledge hinders support for policies aimed at reducing SUP use, as discussed by [25]. The survey results the broader literature suggesting that, increased knowledge about environmental issues encourages more responsible behavior, as seen in [26]. Targeted educational campaigns and legislative support are crucial in addressing knowledge gaps and promoting sustainable practices.

**Table 2:** Consumers Knowledge on Single-use Plastics.

Variable	Frequency (n = 198)	Percent (%)
**Definition of single-use plastics (SUP)**		
Plastics that can be reused multiple times	58	29.3
Plastics designed for one-time use and then thrown away	72	36.4
Plastics that are biodegradable	28	14.1
Recyclable plastics	40	20.2
**The item is most likely considered a SUP**		
Reusable water bottle	81	41.0
Plastic grocery bag	88	44.4
Stainless steel straw	20	10.1
Glass jar	9	5.0
**Ture statements about SUP**		
They are designed to be reused multiple times.	45	23.0
They are typically made from easily biodegradable materials.	84	42.4
They are often discarded after a single use.	56	28.3
They are all readily recyclable in most communities.	13	7.0
**SUP pollution is a major environmental concern due to**		
They are a valuable source of energy.	32	16.2
They break down quickly and harmlessly.	33	17.0
They can persist in the environment for a long time.	111	56.1
They are essential for maintaining food hygiene.	22	11.1
**Heard of microplastics before this survey**		
No	89	45.0
Yes	109	55.1
**Single-use plastics biodegradable**		
No	56	28.3
Not sure	64	32.3
Yes	78	39.4
**Meaning of “Reduce, Reuse, Recycle” in relation to plastic waste management**		
Separate all plastic waste for recycling.	45	23.0
Minimize plastic use by carrying reusable bags and bottles.	44	22.2
Burn plastic waste to reduce landfill volume.	17	9.0
Recycle all plastic waste after a single use.	92	47.0
**Reasons SUP are considered a major environmental concern**		
They are readily biodegradable and break down quickly.	89	45.0
They are easily recycled and turned into new products.	36	18.2
They are not easily broken down and can persist in the environment for a long time.	59	30.0
They are a valuable source of energy when burned.	14	7.0
**Most accurate statements about SUPs**		
They are a safe and healthy alternative to reusable containers.	68	34.3
They are a significant contributor to plastic pollution in Ghana.	105	53.1
They are always clearly labeled for proper disposal.	16	8.1
They are essential for maintaining food hygiene standards.	9	6.0
**Meaning of the recycling symbol on a plastic product**		
The plastic is safe for food contact.	61	31.0
The plastic can be effectively recycled in Ghana.	56	28.3
The plastic is made from recycled materials.	38	19.2
The plastic is biodegradable and will break down quickly.	43	22.0

***Data Source: July 1 to July 31, 2024.***

#### Concerns of consumers about the harmful effects of single-use plastics.

Results in (Table 3) revealed that 55.0% of respondents had minimal concerns about single-use plastic (SUP) waste, 37.3% expressed high concerns, and 8.1% had moderate concerns. This low level of concern is troubling given the environmental impact of SUPs, which make up about 49.0% of global plastic production [27]. Studies, such as those by [21,28], emphasize the need for environmental education to increase awareness and responsibility. Despite the low concern, 37.3% of participants acknowledged the severity of the issue, suggesting that increased awareness could lead to greater public support for legislative actions on plastic waste. The concept of pluralistic ignorance [29] may also explain the low concern, where individuals mistakenly believe others are more accepting of SUP use, hindering collective action. [25] highlight the importance of understanding consumer perceptions for developing effective waste management strategies. Overall, the findings suggest a disconnect between awareness and concern, underscoring the need for interventions that not only educate but also motivate individuals to adopt more sustainable behaviors.

**Table 3:** Consumer Concern about the Harmful Effects of Single-Use Plastics.

Variable	Frequency (n = 198)	Percent (%)
**Aware of the role of single-use plastics (SUP) in air pollution through incineration**		
Yes, very aware	61	31.0
Aware	25	13.0
Somewhat aware	50	25.3
Not very aware	38	19.2
Not aware at all	24	12.1
**Level of concern about the impact of SUPon air quality and pollution**		
Not concerned at all	51	26.0
Slightly concerned	67	34.0
Moderately concerned	28	14.1
Very concerned	40	20.2
Extremely concerned	12	6.1
**Level of concern about the impact of SUP on waterways**		
Not concerned at all	44	22.2
Slightly concerned	70	35.4
Moderately concerned	30	15.2
Very concerned	41	21.0
Extremely concerned	13	7.0
**Level of concern about the impact of SUP on marine ecosystems and wildlife**		
Not concerned at all	55	28.1
Slightly concerned	60	30.3
Moderately concerned	29	15.0
Very concerned	39	20.0
Extremely concerned	15	8.0

***Data Source: July 1 to July 31, 2024.***

#### Consumer perception of single-use plastics’ harmful effects.

Results in (Table 4) revealed that 57.1% of respondents had a good perception of the harmful effects of single-use plastics (SUPs), while 43.0% had a poor perception. Regarding the environmental threat of SUPs, 43.0% viewed it as a significant threat, with 32.3% acknowledging it as a moderate threat. Specifically, 56.0% of participants considered SUPs a moderate to significant threat to marine life. These findings align with studies highlighting growing public awareness of plastic pollution. [25,30] emphasize the importance of consumer perceptions in driving behavior changes and supporting policy actions. The survey’s results reflect the broader scientific consensus on the environmental risks of SUPs, including the contribution to marine debris and the harmful effects on marine ecosystems, as noted by [31,32]. The concern for marine life is further supported by studies documenting the ingestion of microplastics by marine organisms and the toxicity of leachate from SUPs [33,34]. Overall, the survey indicates strong public awareness of the environmental threats posed by SUPs.

#### Consumer practices towards single-use plastic use.

Results in (table 5) reveal concerning consumer behavior regarding single-use plastics (SUPs), with 77.0% of participants purchasing plastic bottled drinks daily, 70.2% buying snacks in plastic packaging, 59.1% using plastic shopping bags, and 63.0% buying takeaway food in Styrofoam packs. These practices indicate a heavy reliance on plastic packaging, which is also noted in the literature as a significant environmental issue [25]. While many respondents reuse plastic items, such as bottles and shopping bags, this behavior does not fully mitigate environmental harm, as reusing plastics can still pose risks like chemical leaching and microplastic contamination [35]. The study also reflects that consumer habits, driven by convenience, play a major role in plastic consumption, as highlighted by [36,37]. The findings suggest that raising awareness alone is insufficient, and interventions are needed to encourage shifts in consumption patterns and reduce plastic waste.

**Table 5:** Consumer Practices towards single-use plastics.

Variable	Frequency (n = 198)	Percent (%)
**Frequency of buying water and soft drinks in plastic bottles**		
Rarely	5	3.0
Daily	152	77.0
Weekly	37	19.0
Monthly	4	2.0
**Frequency of acquiring snacks, bread, sugar, and groundnuts wrapped with plastic films**		
Rarely	8	4.0
Daily	139	70.2
Weekly	44	22.2
Monthly	7	4.0
**Frequency of using plastic shopping bags**		
Rarely	12	6.1
Daily	117	59.1
Weekly	52	26.3
Monthly	17	9.0
**Frequency of buying takeaway food packaged in Styrofoam packs**		
Rarely	10	5.1
Daily	124	63.0
Weekly	50	25.2
Monthly	14	7.1
**Frequency of drinking water from sachet bags**		
Rarely	6	3.0
Daily	135	68.2
Weekly	50	25.3
Monthly	7	4.0
**Number of times of using plastic bottle before disposing of it on average**		
1 time	76	38.4
2-3 times	97	49.0
4-5 times	13	7.0
More than 5 times	12	6.0
**Number of times of using a plastic film before disposing of it on average**		
1 time	88	44.4
2-3 times	77	39.0
4-5 times	20	10.1
More than 5 times	13	7.0
**Number of times using a plastic bag before disposing of it on average**		
1 time	83	42.0
2-3 times	85	43.0
4-5 times	17	9.0
More than 5 times	13	7.0
**Number of times of using a Styrofoam pack before disposing of it on average**		
1 time	87	44.0
2-3 times	84	42.4
4-5 times	17	9.0
More than 5 times	10	5.1

***Data Source: July 1 to July 31, 2024.***

#### Attitudes of consumers towards single-use plastics.

Results in (Table 6) reveal that 51.0% of respondents had a positive attitude toward addressing single-use plastic (SUP) waste, while 49.0% had a poor attitude. Over half (53.1%) recognized SUPs as a major contributor to plastic pollution in Ghana, and 90.4% supported stricter government regulations on SUPs. Additionally, 67.0% agreed that companies should be held responsible for the environmental impact of their SUP products. These findings reflect growing consumer awareness and concern, aligning with global trends and studies such as [38], which highlight the significant role of plastic packaging in global waste. Support for stricter regulations and corporate accountability mirrors findings revealed by [39,40], who emphasize the need for better waste management policies and innovative solutions. The survey also suggests that consumers are increasingly advocating for change, with a recognition of the importance of both government action and corporate responsibility. However, the nearly equal split in attitudes underscores the need for ongoing education and awareness campaigns to foster a culture of responsibility and environmental stewardship.

**Table 6:** Consumer attitudes towards Single-Use Plastics.

Variable	Frequency (n = 198)	Percent (%)
**Frequency of witnessing single-use plastic (SUP) litter on streets or sidewalks in the community**		
Never	11	6.0
Rarely	11	6.0
Sometimes	70	35.4
Often	28	14.1
Always	78	39.3
**Importance of reducing the use of SUP**		
Not important at all	17	9.0
Somewhat important	29	15.0
Moderately important	51	26.0
Very important	76	38.4
Extremely important	25	13.0
**Willingness to pay a small deposit on beverage bottles if money will be refunded when you recycle them**		
No, not at all	31	16.0
Maybe, depending on the deposit amount	80	40.4
Yes, I would be happy to	87	44.0
**Individuals have a responsibility to reduce their use of SUPs**		
No	11	6.0
Maybe, depending on the situation	78	39.4
Yes, to some extent	52	26.3
Yes, absolutely	57	29.0
**Likelihood of switching to reusable alternatives if they were readily available and affordable**		
Not likely at all	15	8.0
Somewhat unlikely	42	21.2
Neutral	27	14.0
Somewhat likely	60	30.3
Very likely	54	27.3
**Reducing the use of SUPs is important**		
Yes, very important	82	41.4
Yes, somewhat important	73	37.0
No, not important	32	16.2
Not sure	11	6.0
**Typically carries a reusable shopping bag when going to the market or shops**		
Never	20	10.1
Rarely	24	12.1
Sometimes	100	51.0
Often	16	8.1
Always	38	19.2
**Typically rinses and separates plastic waste from other household waste for potential recycling**		
Never	35	18.0
Rarely	19	10.0
Sometimes	92	47.0
Often	19	10.0
Always	33	17.0
**Willing to pay small deposit on plastic bottles if it meant you could get your money back when you return them for proper recycling**		
No	14	7.1
Maybe, depending on the deposit amount	98	46.0
Yes	86	43.4
**Willing to pay more for environmentally friendly alternatives to single-use plastics**		
No, not at all	18	9.1
Maybe, depending on the price difference	50	25.3
Yes, if the price difference is reasonable	49	25.0
Yes, I would always choose sustainable packaging	81	41.0
**Supports local regulations aimed at reducing SUP consumption**		
No	17	9.0
Not Sure	58	29.3
Yes	123	62.1
**Importance of reducing SUP footprint**		
Not important at all	58	29.3
Somewhat important	44	22.2
Moderately important	38	19.2
Very important	49	25.0
Extremely important	9	5.0
**Importance of reducing one’s own single-use plastic consumption**		
Not important at all	48	24.2
Somewhat important	51	26.0
Moderately important	38	19.2
Very important	51	26.0
Extremely important	10	5.0
**Likelihood to switch to refillable containers for household products if readily available**		
Not likely at all	22	11.1
Somewhat unlikely	38	19.2
Neutral	43	22.0
Somewhat likely	61	31.0
Very likely	34	17.2
**Know of any regulation on SUPs in Ghana**		
No	66	33.3
Yes	132	67.0
**Supports local regulations aimed at reducing SUPs consumption**		
No	37	19.0
Yes	161	81.3
**Government should implement stricter regulations on SUPs**		
No	19	10.0
Yes	179	90.4
**Effectiveness of SUPs regulations**		
Not effective at all	12	6.1
Somewhat effective	28	14.1
Moderately effective	44	22.2
Very effective	76	38.4
Extremely effective	31	16.0
Others	7	4.0
**Effectiveness of current waste management practices in addressing plastic waste in Ghana**		
Not effective at all	35	18.0
Somewhat effective	36	18.2
Moderately effective	55	28.0
Very effective	51	26.0
Extremely effective	21	11.0
**Level of agreement with the statement “Everyone has a responsibility to reduce plastic waste in Ghana**		
Strongly disagree	19	10.0
Disagree	20	10.1
Neutral	36	18.2
Agree	82	41.4
Strongly agree	41	21.0
**There should be stricter regulations on the use and disposal of SUPs to protect the environment**		
Yes, strongly agree	51	26.0
Yes, agree	83	42.0
Neutral	37	19.0
Disagree	17	9.0
Strongly disagree	10	5.0
**Willing to pay more for products that use sustainable, non-single-use packaging to reduce environmental harm**		
Yes, definitely	74	37.4
Yes, maybe	73	37.0
No, probably not	39	20.0
No, not	12	6.0
**The government should implement stricter regulations on single-use plastics**		
Strongly Agree	153	77.3
Somewhat Agree	25	13.0
Neither Agree nor Disagree	14	7.1
Somewhat Disagree	3	2.0
Strongly Disagree	3	2.0
**Companies should be held responsible for the environmental impact of their SUP products**		
Strongly Agree	132	67.0
Somewhat Agree	46	23.2
Neither Agree nor Disagree	13	7.0
Somewhat Disagree	5	3.0
Strongly Disagree	2	1.0
**I try to avoid products with excessive single-use plastic packaging.**		
Strongly Agree	142	72.0
Somewhat Agree	34	17.2
Neither Agree nor Disagree	15	8.0
Somewhat Disagree	5	3.0
Strongly Disagree	2	1.0
**Education and awareness campaigns can influence consumer behavior and reduce single-use plastic consumption.**		
Strongly Agree	148	75.0
Somewhat Agree	34	17.2
Neither Agree nor Disagree	12	6.0
Somewhat Disagree	2	1.0
Strongly Disagree	2	1.0
**I am willing to pay more for products with biodegradable or sustainable packaging.**		
Strongly Agree	145	73.2
Somewhat Agree	30	15.2
Neither Agree nor Disagree	14	7.1
Somewhat Disagree	1	1.0
Strongly Disagree	8	4.0

***Data Source: July 1 to July 31, 2024.***

#### Consumer disposal methods for single-use plastics.

Results in (Table 7) reveals concerning disposal practices for single-use plastics (SUPs), with 57.0% of respondents discarding plastic bottles in bins, 26.0% burning them, and only 10.1% recycling. These trends align with global issues in plastic waste management, such as the prevalent use of incineration, which releases harmful pollutants, and low recycling rates, which globally remain under 10% [41]. The study also identified barriers to reducing SUP consumption, including a lack of alternatives (35.4%) and awareness (29.3%) about their environmental impact. Economic factors, such as the high cost of reusable alternatives, and the inconvenience of recycling, also hinder sustainable behaviors, as noted in other studies [42].

**Table 7:** Consumer Disposal Methods of single-use plastics.

Variable	Frequency (n = 198)	Percent (%)
**Method of disposing single-use plastic bottles**		
Bin	112	57.0
Burn	51	26.0
Recycle	20	10.1
Leave on the street	11	6.0
Others	4	2.0
**Method of disposing of plastic films**		
Bin	113	57.1
Burn	47	24.0
Recycle	25	13.0
Leave on the street	9	5.0
Others		
**Method of disposing of plastic bags**		
Bin	112	57.0
Burn	43	22.0
Recycle	28	14.1
Leave on the street	10	5.1
Others	5	6.0
**Method of disposing of Styrofoam packs**		
Bin	105	53.0
Burn	53	27.0
Recycle	25	13.0
Leave on the street	11	6.0
Others	4	2.0
**Method of disposing of sachet bags for water?**		
Bin	112	57.0
Burn	51	26.0
Recycle	20	10.1
Leave on the street	10	5.1
Others	5	3.0

***Data Source: July 1 to July 31, 2024.***

## Conclusion

The study highlights that single-use plastic (SUP) pollution is a significant issue in three coastal areas of Accra, with a notable knowledge gap about SUPs’ environmental impact among residents. While daily SUP use is prevalent, there is strong support for stricter government regulations and local initiatives to reduce consumption. However, challenges like the lack of affordable alternatives and insufficient recycling infrastructure persist. The findings suggest that long-term community engagement, increased awareness, and addressing practical barriers can lead to positive changes in attitudes and behaviors. A comprehensive approach, including public education, supportive policies, better waste management, and accessible alternatives, is essential to reduce SUP pollution in these communities.

## Recommendation

The study recommends four key solutions to tackle single-use plastic (SUP) pollution in Accra’s coastal areas. First, public education campaigns should be implemented to raise awareness about the environmental impacts of SUPs, targeting all age groups and correcting misconceptions. Second, enhancing recycling infrastructure through public-private partnerships can improve recycling rates, with initiatives like more recycling bins, deposit-return schemes, and community recycling centers. Third, there is need to enforce policies on SUP use and have stricter regulations, such as bans on certain SUP items and mandatory fees for plastic bags, supported by monitoring efforts. Lastly, promoting affordable, reusable alternatives through subsidies can help overcome barriers to reducing SUP use.

## Limitations of the study

The study on single-use plastic pollution in Korle-Gonno faced several limitations. Community reluctance to participate, and payment requests hindered data collection. Additionally, time constraints limited the ability to gather comprehensive data or conduct follow-up interviews, possibly leading to the underrepresentation of certain demographic groups. Language barriers may have further affected data accuracy, particularly among non-English speakers. These limitations suggest the need for future studies to have longer timelines, better funding, and multilingual research teams for more representative results.
